# Hyaluronan and Its Interactions With Immune Cells in the Healthy and Inflamed Lung

**DOI:** 10.3389/fimmu.2018.02787

**Published:** 2018-11-29

**Authors:** Pauline Johnson, Arif A. Arif, Sally S. M. Lee-Sayer, Yifei Dong

**Affiliations:** ^1^Department of Microbiology and Immunology, Life Sciences Institute, University of British Columbia, Vancouver, BC, Canada; ^2^Department of Clinical Neurosciences, University of Calgary, Calgary, AB, Canada

**Keywords:** hyaluronan, inflammation, fibrosis, macrophages, lung, wound healing, extracellular matrix

## Abstract

Hyaluronan is a hygroscopic glycosaminoglycan that contributes to both extracellular and pericellular matrices. While the production of hyaluronan is essential for mammalian development, less is known about its interaction and function with immune cells. Here we review what is known about hyaluronan in the lung and how it impacts immune cells, both at homeostasis and during lung inflammation and fibrosis. In the healthy lung, alveolar macrophages provide the first line of defense and play important roles in immunosurveillance and lipid surfactant homeostasis. Alveolar macrophages are surrounded by a coat of hyaluronan that is bound by CD44, a major hyaluronan receptor on immune cells, and this interaction contributes to their survival and the maintenance of normal alveolar macrophage numbers. Alveolar macrophages are conditioned by the alveolar environment to be immunosuppressive, and can phagocytose particulates without alerting an immune response. However, during acute lung infection or injury, an inflammatory immune response is triggered. Hyaluronan levels in the lung are rapidly increased and peak with maximum leukocyte infiltration, suggesting a role for hyaluronan in facilitating leukocyte access to the injury site. Hyaluronan can also be bound by hyaladherins (hyaluronan binding proteins), which create a provisional matrix to facilitate tissue repair. During the subsequent remodeling process hyaluronan concentrations decline and levels return to baseline as homeostasis is restored. In chronic lung diseases, the inflammatory and/or repair phases persist, leading to sustained high levels of hyaluronan, accumulation of associated immune cells and an inability to resolve the inflammatory response.

## Hyaluronan in the healthy lung

### Hyaluronan (HA) in lung development

HA is a high molecular mass glycosaminoglycan (>1 MDa) composed of repeating disaccharide units of D-glucuronic acid and N-acetyl glucosamine (1). During fetal development of the lung, HA is present in the interstitium (2) and the alveolar space is filled with amniotic fluid that is rich in HA and hyaladherins that possess anti-inflammatory and wound healing properties (3, 4). During this time, fetal monocytes populate the mouse lung where they differentiate into CD11b^+^ CD11c^lo^ Siglec F^lo^ pre-alveolar macrophages (pre-AMs) (5–7). At birth, air fills the lungs, and pre-AMs develop into functional AMs (CD11c^+^ Siglec F^hi^ CD11b^−^), coinciding with the decrease in HA levels in the lung (7). AMs express high levels of CD44, a cell surface receptor for HA that is required for HA uptake *in vitro* (8, 9), and AMs are responsible for reducing HA levels *in vivo* (2).

### HA expression in healthy lung tissue

**Figure 1:**
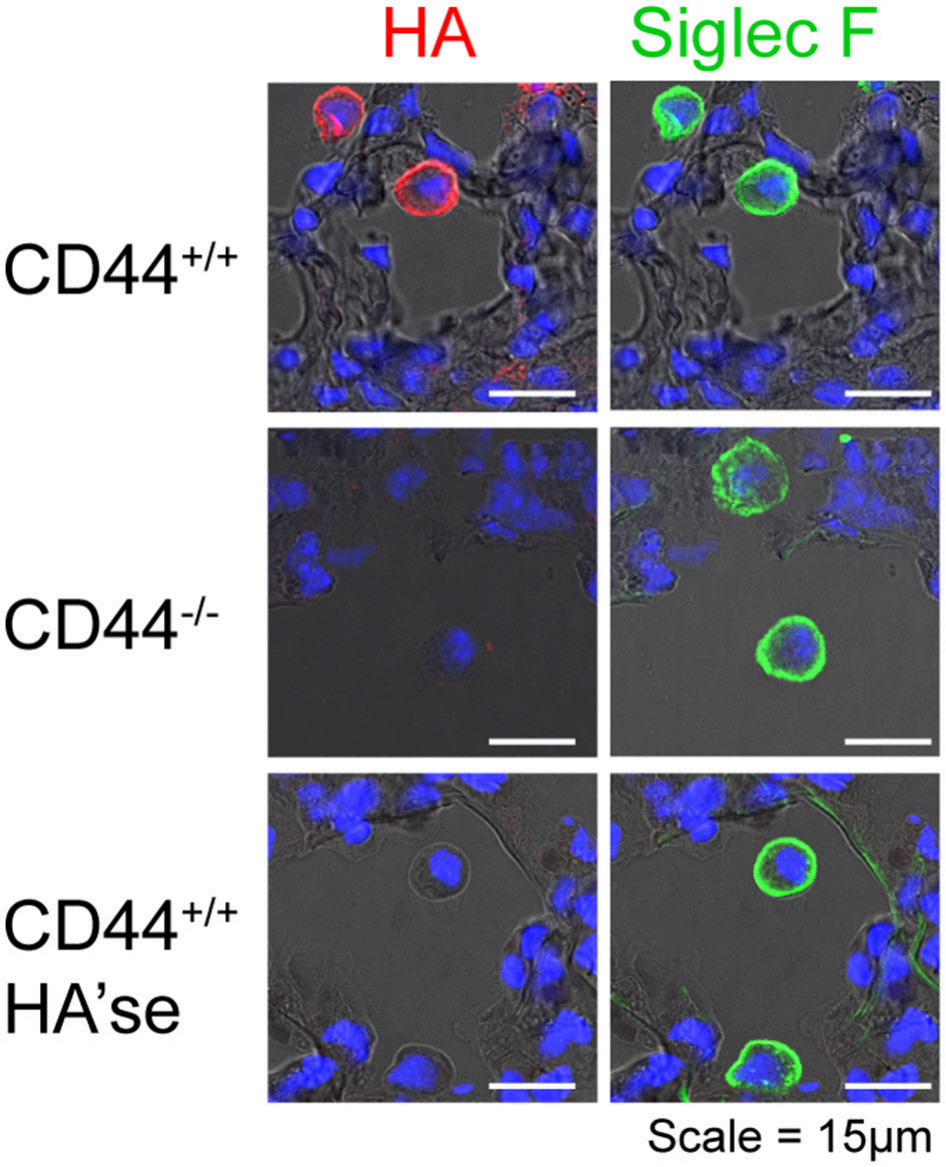
CD44^+/+^ AMs, but not CD44^−/−^ AMs, have a HA coat. Frozen lung sections from CD44^+/+^ and CD44^−/−^ mice were obtained and labeled with HABP to detect HA (red), and Siglec F, a marker of AMs (green), and cell nuclei identified by DAPI (blue). In the lower panel, the sections were treated with bovine testicular hyaluronidase (HA'se). Top and bottom row are lung sections from CD44^+/+^ mice, and the middle row is from CD44^−/−^ mice. Images were captured on a Leica SP5 scanning laser confocal microscope, under identical acquisition settings. Images are representative of those published in Dong et al. (12).

**Figure 2:**
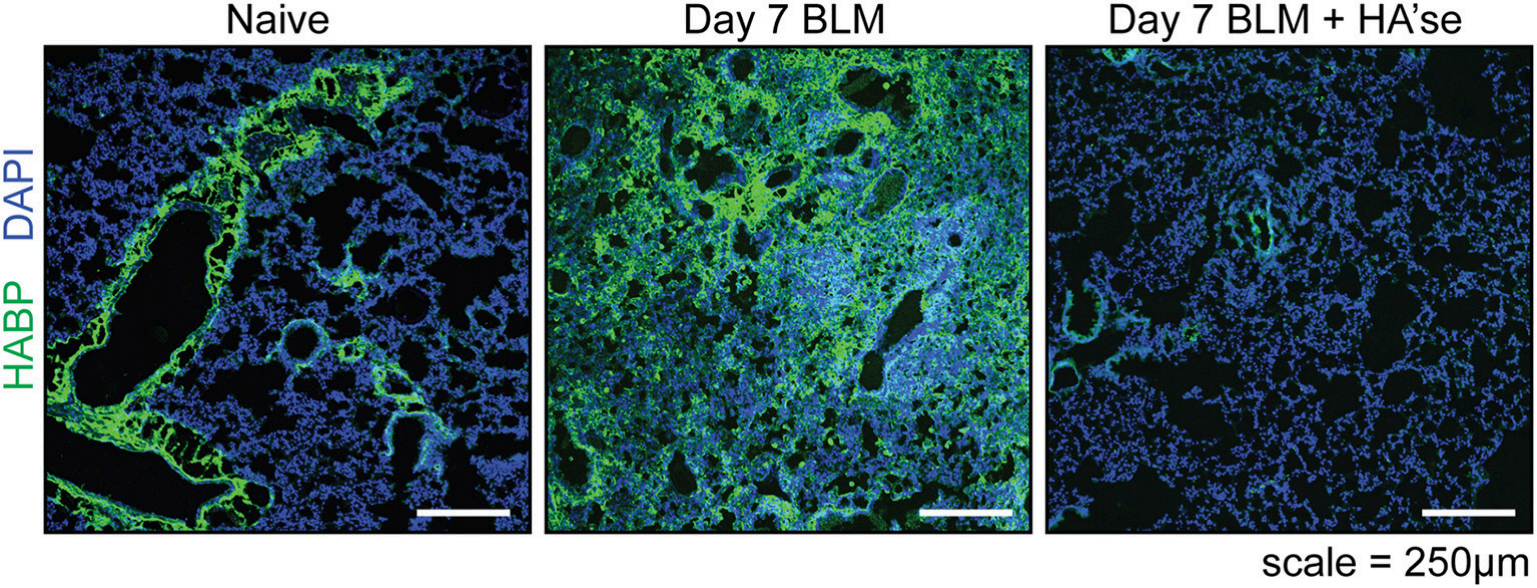
Hyaluronan in the healthy and inflamed mouse lung. Frozen lung sections from CD44^+/+^ mice were labeled with HABP (green) to detect HA, and stained with DAPI (blue) to label cell nuclei. On the left is a representative image of the healthy (naïve) lung where HA is present on the major bronchioles and blood vessels, with little labeling in the interstitium. The middle panel shows HA present in the lung 7 days after bleomycin induced lung inflammation (Day 7 BLM). The panel on the right is a control, showing a lung section from day 7 BLM, after treatment with hyaluronidase. All images were captured using an Olympus FV1000 scanning laser confocal microscope under identical acquisition settings. These images are similar to data described in Cheng et al. (11), Hussell and Bell (13), and Sahu and Lynn (14).

In the uninflamed lung, HA, detected by biotinylated HA binding protein (HABP), is bound to the surface of AMs in the alveolar space [Figure 1 and (10–12)]. HA is also in the basement membrane region of bronchial and bronchiolar epithelium, and in the perivascular region (prominent in the adventitia) of large blood vessels [Figure 2 and (2, 10–12)]. In lung sections, HA is not apparent in the alveolar interstitium or on alveolar epithelium (10). CD44 is present on the basolateral surface of bronchial epithelium (10), consistent with the localization of HA to the basement membrane.

### HA turnover in the alveolar space

In general, HA turns over very rapidly compared to other extracellular matrix components: approximately a third of the body's HA turns over daily (15). HA is produced by HA synthases (HAS1-3) at the plasma membrane that extrude HA into the extracellular space (16). Type II alveolar epithelial cells (AECs) express HAS2 and surface HA (17). HA is loosely attached and shed from the apical surface of primary AEC cultures, and can be observed above the airway epithelium (18), although this is not always the case (10, 12). This suggests that HA can be released into the alveolar fluid above the AECs. Since HA levels are low in bronchoalveolar lavage fluid (BALF) from healthy animals (19), HA must be turned over, possibly by AMs, which take-up and degrade HA *in vitro* (8, 9) and bind HA *in vivo* (12). Clearance of HA involves its degradation into smaller fragments by hyaluronidases such as Hyal 2 (20, 21), TMEM2 (22), and possibly KIAA1199 (23) at the cell surface. These fragments are then internalized by receptors such as CD44 and HARE/Stabilin-2 and taken to the lysosome where they are degraded by Hyal 1 (16, 24, 25).

### HA binding to AMs promotes their survival and maintenance

The ability of CD44 to bind HA is highly regulated in cells (26–30). AMs express a form of CD44 that constitutively binds HA (8, 12, 31). In contrast, CD44 on unactivated monocytes, macrophage colony stimulating factor-derived macrophages, and peritoneal macrophages, do not bind fluoresceinated HA (FL-HA) (31, 32). However, when peritoneal macrophages are introduced into the lung airways, they gain the ability to bind FL-HA (12), highlighting the influence of the alveolar environment on HA binding. Granulocyte-macrophage colony stimulating factor (GM-CSF or CSF-2) and PPARγ are both important in the alveolar space for AM development and maintenance (6, 33) and treatment of bone marrow-derived macrophages with GM-CSF and a PPARγ agonist, rosiglitazone, induces CD44-dependent HA binding (12), implicating these factors in regulating HA binding by AMs in the alveolar space.

AMs possess a HA coat that is anchored to its surface by CD44, and is absent in CD44^−/−^ AMs, Figure 1 and (12). The HA coat was unexpected, given the AMs ability to take-up and degrade HA (8, 9). Although high molecular mass HA (HMW-HA, >1 MDa) predominates in uninflamed lung tissue (19), the size and origin of HA in the AM coat is not known. What is known is that this HA coat promotes the survival of AMs, and its removal by hyaluronidases induces apoptosis (12). CD44^−/−^ AMs are more susceptible to apoptosis and mice lacking CD44 have reduced numbers of AMs in the lung (12). The engagement of HA by CD44 is required for optimal AM survival *in vivo*, as its disruption with an HA blocking CD44 antibody leads to reduced numbers of AMs (12).

### Effect of type II AEC generated HA

AMs reside in the alveolar space, above the AEC layer, in the fluid surfactant layer, where some AMs closely associate with AECs (10, 34). They form intimate connexin-43-dependent gap-junction interactions which can modulate inflammation (34). CD200-CD200R and αvβ6-tumor growth factor beta (TGFβ)-TGFβR interactions further support an association between these cells, which acts to limit AM activation (13, 35). Although type II AECs express HAS2 (17), it is unclear if type I AECs, which form the majority of the alveolar epithelial surface, also synthesize HA. At homeostasis, HA produced by type II AECs may be bound and/or taken up by AMs, keeping HA levels low in the surfactant layer. Alternatively, HA binding by AMs may strengthen their immunosuppressive connection with AECs, or independently promote immunosuppressive behavior, as HMW-HA has been shown to limit activation in other cells (36, 37). HAS2 overexpression in type II AECs protects these cells against bleomycin-induced apoptosis, as does HMW-HA (38). Conversely, loss of HAS2 expression leads to their decreased renewal capacity *in vitro* (17) supporting the idea that, like AMs, HA promotes the survival/self-renewal ability of type II AECs. HMW-HA also supports the survival/ self-renewal capacity of stem cells [reviewed in (39–41)], suggesting a common function for HMW-HA in promoting the survival of cells capable of self-renewal.

## Ha in the inflamed lung

### HA levels are elevated in lung disease

HA is upregulated during tissue inflammation in many diseases and across many tissues (42, 43). The upregulation of HA is a general characteristic of inflammation, occurring in a broad repertoire of inflammatory and infectious conditions irrespective of the type of stimuli or the type of immune response generated (inflammatory: type 1, or allergic, fibrotic: type 2), and has been the subject of many excellent reviews (43–47).

In the lung, the concentration of HA is elevated in the BALF of patients suffering from asthma (14), chronic obstructive pulmonary disease (48), interstitial pulmonary fibrosis (49), and other lung diseases [reviewed in (43, 44)]. In animal models, HA is upregulated in the bleomycin model of sterile injury (19, 50), asthma (ova and cockroach allergen) (11, 51), ozone-induced airway hyperreactivity (52), LPS-induced acute lung injury, and *Escherichia coli* (53), *Klebsiella pneumoniae* (54), or Influenza infection (55).

### Hyaladherin expression is increased upon inflammation

In addition to HA, several hyaladherins are upregulated upon lung inflammation [reviewed in (46, 56–60)]. These include versican, the heavy chain (HC) of the inter-alpha-trypsin inhibitor (IαI), link protein, tumor necrosis factor stimulated gene 6 (TSG-6), pentraxin-3, and aggrecan, which can bind to, and modify, the HA glycocalyx. TSG-6 is an enzyme that catalyzes the covalent transfer of the HC of IαI to HA (59). TSG-6 can also independently bind and crosslink HA to form a more compact matrix (61) that has increased binding to CD44 (62). In intestinal inflammation, smooth muscle cells generate distinctive HA cables that are modified by HC and adhesive for platelets, key cells in the wound healing process (63). Activated platelets express Hyal 2 (64) and can degrade HA cables down to 20 kDa fragments, but lack Hyal 1 which would allow complete digestion (65).

### HA levels correlate with inflammatory infiltrate

Animal models of lung infection, injury, and inflammation allow a closer analysis of the changes that occur to HA during the inflammatory response. In models of acute and chronic asthma, HAS1 and 2 are rapidly upregulated in the lung only a few hours after re-exposure to allergen, while Hyal 1 and 2 decrease over time, leading to the accumulation of HA that is maximal after 6 days and is maintained with continued chronic stimulation (11). Inflammatory stimuli also induce TSG-6 expression, which maintains HA deposition and eosinophil recruitment (66). Eventually, HA levels return to baseline after about 8 weeks (11).

In a model of acute sterile lung inflammation, a single dose of bleomycin induces HA expression in the lung tissue (67) and Figure 2 (Day 7 BLM), which, together with leukocyte infiltration, peaks at day 7 (19, 50). In inflammation, HA has a smaller average molecular mass of 0.5 MDa compared to 1.5 MDa in naïve lungs (19). Detection of HA decreases as collagen deposition increases in the remodeling phase (day 14 to past 21) (19, 47). Final resolution of the response involves the return of HA to baseline levels which occurs around 5 weeks (50), together with the removal of collagen, myofibroblasts, fibrotic macrophages, and the restoration of lung epithelium by type II AECs (24).

In the absence of CD44, HA levels continue to increase after bleomycin treatment and the severity of inflammation increases. HA sizes become smaller and more heterogeneous, ranging from 0.02 to 2 MDa (19). HA levels and the leukocyte infiltrate are reduced if CD44^+/+^ bone marrow cells are transplanted into irradiated CD44^−/−^ mice, implicating CD44^+/+^ leukocytes (potentially macrophages) in the uptake and clearance of HA.

HMMR/RHAMM has been described as a receptor for HA-mediated motility and as a intracellular centrosomal protein involved in spindle orientation and integrity that is upregulated during the cell cycle (68). Genetic deletion of HMMR at exon 2 is lethal in mice (69) whereas deletion of exons 8 or 10 leads to their survival (70, 71). The exon 8 targeted mice have reduced HA and lung inflammation and less inflammatory macrophages in response to bleomycin, whereas mice overexpressing RHAMM in scavenger receptor A positive macrophages show the opposite (72). Thus the effects of RHAMM are distinct from that of CD44. RHAMM affects both macrophage proliferation and motility *in vitro* (72), but further work is required to determine its mechanism in lung inflammation.

Influenza virus causes severe damage in the lung that is repaired for months after the virus has been cleared (35). During this recovery period, the lung is more susceptible to bacterial infections. Recent work found that HA levels remain high in the lung tissue due to elevated HAS2 expression in epithelial, endothelial, and fibroblast cells (55). TSG-6 levels are also elevated and this generates a HC-modified HA matrix (55). Interestingly, a single dose of hyaluronidase at day 6 after influenza infection reduces the HA content in the lung, reduces the number of F4/80^+^CD11b^+^CD11c^lo^ macrophages and improves lung function at day 16 (55).

These models of lung inflammation show that HA levels increase with inflammation, suggesting a role for HA in supporting the leukocyte infiltrate. HA is hygroscopic and has been linked with edema formation (73), which would allow easier movement of leukocytes in the damaged tissue. In the bleomycin model, HA levels decrease after the peak of inflammation, whereas in the asthma and influenza models, increased HA levels persist. Only after remodeling and completion of the repair process do HA levels return to baseline levels.

### HA fragments in inflammation: present challenges

It is important to keep in mind that both pericellular and extracellular HA matrices are thought to turnover frequently. This means that HA is continually synthesized and degraded, and during inflammation, increased synthesis leads to the accumulation of HA. The size of this HA is more heterogenous and HA fragments (varying from small oligosaccharides to 0.5 MDa) are considered damage-associated molecular patterns that stimulate inflammatory responses [reviewed in (26), (36), (38), (42), (43)]. However, this has recently been challenged by studies showing that some HA and hyaluronidase preparations are contaminated with endotoxin (74, 75). This, together with the absence of evidence showing direct binding of HA fragments to TLRs, has questioned whether HA fragments directly promote inflammation. An alternative explanation is that smaller sized HA fragments displace HMW-HA bound to CD44, and disrupt its protective, immunosuppressive effect (76). In some cells, HMW-HA inhibits NF-κb signaling (77), and so its displacement by HA fragments would result in a proinflammatory NF-κb response. Another explanation for the variation in results seen with different sized HA fragments may arise from the use of polydisperse HA where a range of HA sizes within a single preparation compete for receptors to initiate the inflammatory signal (78). There is some evidence that HARE/Stabilin-2 responds to specific sizes of HA (79). Why would HA fragments as large as 200 kDa be seen differently from 1 MDa HA, when HA receptors, such as CD44, recognize just a few sugar units (80)? The answer may lie in the ability of different forms of HA to cluster HA receptors and thereby influence the signal delivered. Recent work shows that HA undergoes a transition from a random coil to a rod shape at around 200 kDa, suggesting that these forms could differentially impact HA receptor clustering and signaling (81). In support of this idea, TSG-6-crosslinked HA creates a more compact matrix (61) that is more efficiently recognized by the HA receptors, CD44 (62) and Lyve-1 (82). HC-modified HA forms cables (83) that also alter how HA is perceived by the cell (63). Clearly, more work is needed to understand the contribution of HA fragments and hyaladherins in the inflammatory response.

### HA binding immune cells in lung inflammation and repair

In a type I inflammatory response, neutrophils and inflammatory monocytes are recruited to the site of infection, where they contribute to the proinflammatory environment and respond to the threat. Neither of these cell types bind appreciable levels of FL-HA (26). In a type 2 allergic response, eosinophils are recruited (11) and these cells bind low levels of FL-HA (84). In animal models of acute and chronic asthma, eosinophils are present in HA-rich areas of the inflamed lung (11). Once in the inflamed lung, inflammatory monocytes differentiate into macrophages and become F4/80^+^ CD11b^+^ CD11c^+^ Siglec F^lo^ during the repair phase (12, 85). These macrophages bind FL-HA (12), produce TGFβ and drive bleomycin-induced fibrosis (85). In a mouse model of allergic asthma, F4/80^+^ macrophages are found with HA and versican in the subepithelial region of the lung (86). Thus, HA binding may provide one possible means of bringing immune cells such as eosinophils and fibrotic macrophages into close proximity with the HA-producing myofibroblasts involved in repair.

### HA producing myofibroblasts in lung repair

During the course of lung inflammation, the cells responsible for the increase in HA synthesis have not been clearly defined. However, individually, fibroblasts, myofibroblasts, endothelial cells, smooth muscle cells, and type II AECs can all produce pericellular HA coats in response to inflammatory or reparative stimuli (24, 42, 46). Myofibroblasts are major HA-producing cells that have key roles in wound repair, collagen deposition, and fibrosis (24). TGFβ induces the differentiation of fibroblasts into smooth muscle actin positive myofibroblasts and enhances their production of pericellular HA (87). HA further promotes their differentiation and maintenance (85, 88, 89). TGFβ induces HAS1 and 2 expression in fibroblasts (90, 91) and reduces Hyal 1 and 2 expression (92), and this HA is required for TGFβ-induced fibroblast proliferation by providing a late pERK signal (93, 94). TGFβ also induces TSG-6 which generates HC modified HA cables that facilitate myofibroblast differentiation (88, 89, 95). Overexpression of HAS2 in lung myofibroblasts leads to a severe fibrotic response and invasive fibroblast phenotype (96), while the deletion of HAS2 in fibroblasts increases cellular senescence in a mouse model of pulmonary fibrosis (97). Thus, pericellular HA is intimately linked to the fibrotic/repair function of myofibroblasts.

### Type II AECs in lung inflammation and repair

In the repair phase, damaged type I AECs are replaced by the differentiation of type II AECs, which have stem cell-like properties (98). Both the loss and overexpression of HAS2 in type II AECs have significant effects on epithelial cell repair in response to bleomycin, with HA protecting against epithelial damage and apoptosis, and the loss of HA impairing AEC renewal and leading to severe fibrosis (17, 38). Likewise, type II AECs isolated from patients with severe pulmonary fibrosis have reduced levels of surface HA (17). Thus, HA has a protective effect on type II AECs.

### AMs in lung inflammation and repair

Lung inflammation results in the depletion of tissue resident, fetal monocyte-derived AMs, with the extent of their loss proportional to the severity of the insult [(12, 33, 85, 99, 100) and Dong et al., unpublished data]. The cause of this loss is not understood, but it has been suggested that macrophage necrosis triggers the ensuing inflammatory response (101–103). Maximal loss of AMs occurs at the peak of leukocyte infiltration and HA accumulation, after which the AMs increase in numbers, due in large part to self-renewal (12, 33, 100). Since AMs are implicated in HA uptake and degradation (8, 9), it is possible that their loss could contribute to the increased levels of HA observed upon inflammation. However, this remains to be determined. Whether the change in size of HA during inflammation also impacts AM survival, or its uptake by AMs, is also not known. During inflammation, AMs gain CD11b, but still retain high levels of CD11c and Siglec F, as well as their ability to bind HA, and are distinguishable from the newly differentiated monocyte-derived macrophages (CD11c^+^, CD11b^+^, Siglec F^lo^) that play a critical role in driving repair/fibrosis (12). The recovery of AM numbers during the repair phase suggests a function in the later stages of the response, perhaps in helping the return to homeostasis. With the resolution of inflammation and repair, monocyte-derived macrophages become phenotypically identical to tissue resident AMs, but they may not be functionally identical (104, 105).

In summary, HA levels are low in the alveolar space in the healthy lung, and HA bound to AMs promotes their survival. In the lung tissue, HA is present in the basement membranes of bronchioles and in the perivascular area. HA levels dramatically increase upon lung inflammation, perhaps enabling leukocytes to access the site of injury. Hyaladherins are also produced in response to inflammation and their interactions with HA can influence its physical properties and increase immune cell interactions. After repair and remodeling, HA levels eventually return to baseline, as inflammation is resolved. In situations of chronic disease, the persistence of HA is associated with increased inflammatory and fibrotic responses that are not resolved.

## Ethics statement

The figures were taken from work carried out in accordance with the guidelines for ethical animal research from the Canadian Council of Animal Care with protocols approved by the University of British Columbia Animal Care Committee.

## Author contributions

PJ wrote the initial draft which was then worked on by all authors. AA provided the figures.

### Conflict of interest statement

The authors declare that the research was conducted in the absence of any commercial or financial relationships that could be construed as a potential conflict of interest.
